# Chronic Prostate Cancer-Related Symptoms and Toxicities Following Stereotactic Body Radiation Therapy: Implications for Future Radionuclide Therapy

**DOI:** 10.7759/cureus.85034

**Published:** 2025-05-29

**Authors:** Diya Kallam, Kelly Gaudian, Ryan Collins, Zoya Zwart, Min Ji Koh, Malika T Danner, Alan L Zwart, Deepak Kumar, Suy Simeng, Lisa Gudenkauf, Mark Fallick, Giuseppe Esposito, Brian Gonzalez, Sean Collins

**Affiliations:** 1 Radiation Oncology, George Washington University School of Medicine and Health Sciences, Washington, D.C., USA; 2 Radiation Oncology, Washington University School of Medicine, St. Louis, USA; 3 Radiation Oncology, College of William and Mary, Williamsburg, USA; 4 Department of Radiation Oncology, Georgetown University, Washington, D.C., USA; 5 Julius L. Chambers Biomedical/Biotechnology Research Institute, North Carolina Central University, Durham, USA; 6 Department of Radiation Medicine, Georgetown University, Washington, D.C., USA; 7 Radiation Oncology, Moffitt Cancer Center, Tampa, USA; 8 Radiation Oncology, Novartis, East Hanover, USA; 9 Department of Nuclear Medicine, Georgetown University, Washington, D.C., USA; 10 Radiation Oncology, Tampa General Hospital, Tampa, USA

**Keywords:** nuclear medicine, prostate cancer, quality of life (qol), radiation oncology education, radioligand therapy

## Abstract

Background

Men treated for prostate cancer can experience a high symptom burden. Stereotactic body radiation therapy (SBRT) is increasingly utilized in the management of localized prostate cancer, while radionuclide therapy (RNT) is rapidly becoming a standard treatment option for metastatic prostate cancer. The Functional Assessment of Cancer Therapy-Radionuclide Therapy (FACT-RNT) is a newly validated questionnaire assessing symptoms and toxicities among patients receiving RNT. This study aimed to characterize long-term, patient-reported symptoms in prostate cancer survivors treated with SBRT using the FACT-RNT questionnaire, and to explore whether such symptoms may inform risk stratification for future RNT candidacy.

Methodology

We conducted a cross-sectional assessment of symptoms and toxicities after prostate SBRT using the FACT-RNT, a 15-item questionnaire assessing common RNT-related symptoms and toxicities across several domains, including fatigue, salivary/lacrimal gland dysfunction, gastrointestinal (GI) difficulties, and pain. Responses to individual questions were grouped into three clinically relevant categories (i.e., absent (“not at all”), mild (“a little bit” to “somewhat”), moderate to severe (“quite a bit” to “very much”)). Standard error was calculated at a 95% confidence interval. However, there are methodological limits to linking SBRT symptoms directly with RNT tolerance.

Results

Prostate cancer patients (N = 296) who had completed SBRT a median of six years prior (range = 0-13 years) completed the FACT-RNT questionnaire at a response rate of 49.5% (296 of 598 consented patients). Patients completed prostate SBRT at a median of six years before questionnaire administration (range = 0-13 years). The median age of responders was 78 years, 76% of patients were white, and 69% were of intermediate risk disease. Overall, 25% of respondents were mildly (21%) or moderately to severely (4%) bothered by treatment side effects. Respondents endorsed at least mild fatigue (53%), xerostomia (29%), constipation (23%), and pain (20%).

Conclusions

A notable percentage of prostate cancer patients treated with SBRT report toxicities commonly associated with RNT, such as fatigue, salivary/lacrimal gland dysfunction, GI difficulties, and pain. Results of this study underscore the importance of administering the FACT-RNT before RNT treatment to obtain a baseline assessment of symptoms, evaluate pre-treatment risk for RNT toxicities (e.g., salivary gland dysfunction), and better differentiate baseline abnormalities in function from RNT-related side effects.

## Introduction

Radionuclide therapy (RNT) is increasingly utilized as a treatment for metastatic prostate cancer due to its ability to comprehensively address all disease sites [1]. The most commonly utilized target of radionuclide particles is the prostate-specific membrane antigen (PSMA), a protein abundantly expressed by most prostate cancer cells [2,3]. Radioisotopes such as lutetium-177 (Lu-177) are linked to PSMA-binding small molecules [4]. Lu-177 emits low-energy beta particles within a short range, limiting damage to adjacent healthy tissue. Novel agents with shorter-acting, high-energy, alpha-generating actinium-225 (Ac-225) are also currently in development [5].

RNT has been evaluated for efficacy and safety by several studies, which showed a greater than 50% decline in prostate-specific antigen (PSA) in a significant number of patients with acceptable toxicity [6]. Large phase III studies, such as TheraP [6] and VISION [7], found improved response and survival in those treated with RNT compared to standard-of-care or intention-to-treat groups. In the LuPSMA phase II study, common adverse events (any grade, >30%) were dry mouth (66%), nausea (48%), and fatigue (38%). Research is currently ongoing to reduce toxicities and maximize quality of life [8].

PSMA-directed, RNT-related symptoms of salivary/lacrimal gland dysfunction, gastrointestinal (GI) difficulties, fatigue, and pain are likely attributable to PSMA expression in non-malignant tissues, such as the kidneys, salivary glands, and intestinal mucosa [9]. Notably, patients presenting for RNT are often older males with pre-existing symptom burden after prior prostate cancer treatment, such as androgen deprivation therapy (ADT) and/or stereotactic body radiation therapy (SBRT) [10]. Thus, it is important to assess and address treatment-related symptoms and toxicities before RNT. The Functional Assessment of Cancer Therapy-Radionuclide Therapy (FACT-RNT) is a newly validated measure of common RNT-related toxicities and symptoms, such as salivary/lacrimal gland dysfunction, GI difficulties, fatigue, and pain [11]. These include some symptoms not commonly assessed in other measures of prostate cancer-related morbidities. This study aimed to characterize long-term, patient-reported symptoms in prostate cancer survivors treated with SBRT using the FACT-RNT questionnaire, and to explore whether such symptoms may inform risk stratification for future RNT candidacy. Long-term symptoms in survivors of prostate cancer have been previously studied by many, and there are several frameworks, including the Expanded Prostate Cancer Index Composite-26 within the International Consortium for Health Outcomes Measurement framework, that looks at 26 domains of quality of life in prostate cancer survivors who had previously been treated. This study similarly utilizes the FACT-RNT as a framework for assessing symptom burden. There are some methodological limits to linking SBRT symptoms directly with RNT tolerance, given the design of the study.

This article was previously presented as a meeting abstract at the 2024 ASTRO American Society for Radiation Oncology Annual Conference on September 30, 2024.

## Materials and methods

Study cohort

Patients eligible for this cross-sectional study had localized prostate cancer treated with SBRT at Georgetown University Hospital. Beginning in 2012, surveys were sent via postal mail to patients without metastatic progression who consented to participate in a broader, IRB-approved prospective institutional study (approval number: 12-1175). The current study focuses on a cross-section of patient-reported data on toxicities/symptoms collected in 2023. This was consecutive sampling, and the patients were mailed the survey once with no further follow-ups afterwards. We reviewed medical records to collect demographic information on age, race, body mass index (BMI), PSA, digital rectal examination (DRE), and Gleason score. Risk groups were established using the D’Amico classification. The Charlson Comorbidity Index (CCI), assessed using a self-report survey, was calculated to assess patients’ comorbidities before SBRT. Any missing data was not included in the final analysis and treated as a blank or deleted row. There was no threshold for survey completion, and even those who had partially completed the survey were included in the study.

Outcomes

The primary outcome of this study was symptoms/toxicities, as assessed by the FACT-RNT questionnaire (www.facit.org/measures/FACT-RNT), a 15-item measure of common RNT-related symptoms, such as fatigue, salivary/lacrimal gland dysfunction, GI difficulties, and pain (11). Permission to use the questionnaire was obtained from the original authors, who are also authors of this paper. The FACT-RNT is a standardized measure to monitor toxicities among prostate cancer patients in RNT trials and clinical settings. Although the FACT-RNT is used to measure RNT-related side effects, this study repurposes the same questionnaire to see if the long-term survivors of prostate cancer with previous SBRT had any of those side effects to begin with, which may be worsened by RNT. Aside from patients reporting no toxicity (i.e., “not at all”), responses were grouped into two clinically relevant categories, namely, mild (i.e., “a little bit” to “somewhat”) and moderate to severe (“quite a bit” to “very much”).

Exposure

Prostate SBRT was performed as previously described (12). Gold fiducials were placed into the prostate. Fused CT and MRI scans were used for treatment planning. The clinical target volume (CTV) included the prostate and the proximal seminal vesicles. The planning target volume (PTV) equaled the CTV expanded 3 mm posteriorly and 5 mm in all other dimensions. The prescription dose was 35-36.25 Gy to the PTV delivered in five fractions of 7-7.25 Gy.

## Results

Of the 598 patients invited to participate, 296 completed the FACT-RNT (response rate = 49.5%). Patients completed prostate SBRT a median of six years before questionnaire administration (range = 0-13 years). Patient and treatment characteristics are reported in Table 1. The median age of responders was 78 years (51-107). Overall, 76% identified as White, and 17% identified as Black. Nearly half (49%) had a BMI in the overweight range (BMI = 25-29.9 kg/m^2^), and 23% had a BMI in the obese range (BMI ≥ 30 kg/m^2^). The median baseline CCI score for our patient cohort was 0, with only 5% of patients having a CCI score above 3 (Table 1). Most (69%) had intermediate-risk prostate cancer, and 35% of patients received ADT.

**Table 1:** Demographic and clinical characteristics of the study sample. IQR: interquartile range; ADT: androgen deprivation therapy; BMI: body mass index

Characteristics	N (%)
Age (years), median (IQR)	
<70	37/287 (13)
70–79	127/287 (44)
80–90	114/287 (40)
>90	9/287 (3)
Race	
White	216/286 (76)
Black	50/286 (17)
Other	20/286 (7)
ADT	
Yes	99/286 (35)
No	187/286 (65)
BMI (kg/m^2^)	
<18.5	1/205 (1)
18.5–24.9	55/205 (27)
25–29.9	101/205 (49)
30–34.9	38/205 (18)
34.9–39.9	8/205 (4)
40–44.9	2/205 (1)
Comorbidity Index	
None (0)	123/174 (71)
Mild (1–2)	42/174 (24)
Moderate (3–4)	4/174 (2)
Severe (>5)	5/174 (3)

**Figure 1:**
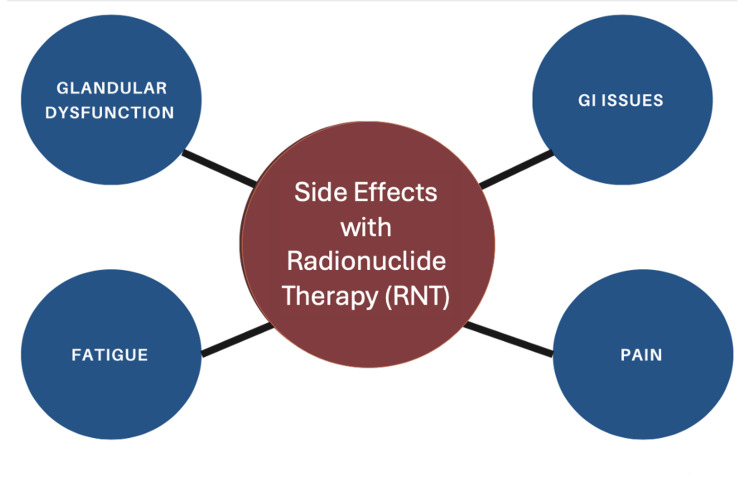
General categories of toxicities/symptoms assessed by the FACT-RNT questionnaire. Image created by Diya Kallam. FACT-RNT: Functional Assessment of Cancer Therapy-Radionuclide Therapy

Frequencies of patient-reported toxicities and symptoms are presented in Table 2. Overall, 25% of respondents were mildly (21%) or moderately to severely (4%) bothered by treatment side effects. Other toxicities and symptoms assessed were grouped into four major categories, i.e., fatigue, salivary/lacrimal gland dysfunction, GI difficulties, and pain (Figure 1).

**Table 2:** FACT-RNT survey results. FACT-RNT: Functional Assessment of Cancer Therapy-Radionuclide Therapy

	Severity score, N (%)		
Side effects	Not at all	A little bit to somewhat	Quite a bit to very much
Dry mouth	210/296 (71)	71/296 (24)	15/296 (5)
Dry eyes	204/296 (69)	77/296 (26)	15/296 (5)
Urination difficulty	195/296 (66)	92/296 (31)	9/296 (3)
Nausea	281/296 (95)	12/296 (4)	3/296 (1)
Vomiting	296/296 (100)	0/296 (0)	0/296 (0)
Diarrhea	252/296 (85)	38/296 (13)	6/296 (2)
Constipation	228/296 (77)	59/296 (20)	9/296 (3)
Loss of appetite	263/296 (89)	30/296 (10)	3/296 (1)
Fatigued	139/296 (47)	127/296 (43)	30/296 (10)
Interference due to fatigue	198/296 (67)	74/296 (25)	24/296 (8)
Pain	237/296 (80)	44/296 (15)	15/296 (5)
Bone pain	252/296 (85)	32/296 (11)	12/296 (4)
Interference due to pain	246/296 (83)	35/296 (12)	15/296 (5)
Bother from chronic symptoms and toxicities	222/296 (75)	62/296 (21)	12/296 (4)
Social isolation due to treatment	272/296 (92)	21/296 (7)	3/296 (1)

The most common patient-reported toxicity/symptom was fatigue. Respondents reported experiencing mild (43%) or moderate to severe (10%) fatigue (Table 2) with mild (26%) or moderate to severe (8%) interference due to fatigue. Regarding salivary/lacrimal salivary/lacrimal gland dysfunction, respondents reported mild (24%) or moderate to severe (5%) dry mouth and mild (26%) or moderate to severe (5%) dry eyes (Table 2). Among GI difficulties, constipation was the most common concern, with 20% of respondents reporting mild constipation, and 3% reporting moderate to severe constipation (Table 2). Diarrhea was less common, with 15% of men reporting at least mild diarrhea. Bothersome nausea, vomiting, and loss of appetite were rare, and fewer than 5% noticed any nausea or vomiting, and fewer than 15% noticed any diarrhea. Overall, pain was reported as mild in 15% of respondents and moderate to severe in 5% of respondents, while bone pain was reported as mild in 11% of patients and moderate to severe in 4% of respondents (Table 2).

## Discussion

This cross-sectional study examined toxicities and symptoms among prostate cancer patients previously treated with SBRT, and no RNT and common RNT-related symptoms, using the FACT-RNT, a measure of common RNT-related toxicities and symptoms. Fatigue was the most commonly reported toxicity/symptom in this sample. Both SBRT and RNT have been associated with fatigue and are more common among the elderly, with negative impacts on quality of life and overall well-being [12]. Importantly, fatigue may serve as a rate-limiting toxicity for patients considering RNT [13], underscoring the importance of assessing fatigue before RNT.

More than one-fourth of the sample reported at least mild xerostomia. Notably, xerostomia is not a common side effect of prostate SBRT [14] and may be more attributable to the older age of our sample [15]. Among patients receiving head and neck irradiation, the incidence and severity of xerostomia were dependent on age, salivary gland dose, and baseline xerostomia severity [16]. The prevalence of xerostomia in this sample raises concerns regarding RNT tolerability, as RNT could further exacerbate existing xerostomia. One prior study indicated the presence of xerostomia in more than 50% of patients after just one RNT infusion and in 100% of patients who received seven infusions [17]. RNT is known to cause issues with mastication, swallowing, and speech, and can exacerbate existing periodontal disease and tooth decay [17], negatively impacting quality of life. RNT-related xerostomia can be managed with sialagogues, such as pilocarpine and cevimeline, which have been tested in head and neck cancer patients [17], although severe xerostomia can be treatment-limiting [18].

At least mild constipation was reported by 23% and diarrhea was reported by 15% of patients in this sample. Although radiation proctitis, including bowel frequency/urgency, incontinence, rectal bleeding, and rectal pain, can present as a common acute side effect of prostate SBRT [19], symptoms mostly resolve within months to years after treatment [19]. This is consistent with our data showing that moderate to severe constipation was reported by only 5% of patients treated with prostate SBRT at a median of six years post-SBRT, presenting a lower risk to RNT tolerance.

Moderate to severe pain was reported in around 5% of participants in this sample and may be attributable to older age or other morbidities [14]. Indeed, underlying chronic pain is reported in up to 50% of elderly individuals and in even greater proportions of individuals living in nursing facilities or other long-term care facilities [10,20]. Our study focused on symptom burden among older men treated with prostate radiotherapy to help inform the burden of toxicities with subsequent treatments such as RNT [21]. However, this is a cross-sectional, retrospective study and does not have predictive power over RNT tolerance, but can help guide future studies and clinical decision-making related to subsequent treatment options such as RNT.

Specific strengths associated with our study include the large sample size, which included specific data regarding treatment course and side effect profiles. Notable study limitations include cross-sectional analysis with patients many years out from SBRT, limiting our ability to discern whether symptoms were treatment-related or related to aging or other morbidities. Additionally, the survey instrument we used was technically targeted toward RNT symptoms only, but we used it for patients who were long-term survivors after receiving SBRT. Lastly, there is a possibility of responder bias given that we had only a 49% response rate.

## Conclusions

The most prevalent and concerning patient-reported symptoms were fatigue and xerostomia, which could negatively impact RNT tolerability. Symptoms commonly attributed to RNT also exist in prostate cancer survivors years after SBRT, and may represent a background burden in aging oncology populations. These findings suggest that baseline symptom assessment may be important when considering cumulative treatment toxicity in sequential therapy paradigms. Future studies should evaluate the impact of baseline xerostomia and fatigue on RNT tolerance.

## References

[1] O'Dwyer E, Bodei L, Morris MJ (2021). The role of theranostics in prostate cancer. Semin Radiat Oncol.

[2] Israeli RS, Powell CT, Corr JG, Fair WR, Heston WD (1994). Expression of the prostate-specific membrane antigen. Cancer Res.

[3] Murphy GP, Elgamal AA, Su SL, Bostwick DG, Holmes EH (1998). Current evaluation of the tissue localization and diagnostic utility of prostate specific membrane antigen. Cancer.

[4] Hennrich U, Eder M (2022). [(177)Lu]Lu-PSMA-617 (Pluvicto(TM)): the first FDA-approved radiotherapeutical for treatment of prostate cancer. Pharmaceuticals (Basel).

[5] Sathekge M, Bruchertseifer F, Vorster M (2020). Predictors of overall and disease-free survival in metastatic castration-resistant prostate cancer patients receiving (225)Ac-PSMA-617 radioligand therapy. J Nucl Med.

[6] Hofman MS, Violet J, Hicks RJ (2018). [177Lu]-PSMA-617 radionuclide treatment in patients with metastatic castration-resistant prostate cancer (LuPSMA trial): a single-centre, single-arm, phase 2 study. Lancet Oncol.

[7] Sartor O, de Bono J, Chi KN (2021). Lutetium-177-PSMA-617 for metastatic castration-resistant prostate cancer. N Engl J Med.

[8] Kuo HT, Zhang Z, Zhang C (2023). Lys-urea-Aad, Lys-urea-Cmc and Lys-urea-Cms as potential pharmacophores for the design of PSMA-targeted radioligands to reduce off-target uptake in kidneys and salivary glands. Theranostics.

[9] Ells Z, Grogan TR, Czernin J, Dahlbom M, Calais J (2024). Dosimetry of [(177)Lu]Lu-PSMA-targeted radiopharmaceutical therapies in patients with prostate cancer: a comparative systematic review and metaanalysis. J Nucl Med.

[10] Buchholz I, Janssen MF (2023). EQ-5D-3L norms for the European older population: country-specific norms for 15 European countires based on the survey of health, ageing, and retirement in Europe. Value Health.

[11] Gudenkauf LM, Chavez MN, Maconi ML (2023). Developing a patient-reported outcome measure for radionuclide therapy for prostate cancer. J Nucl Med.

[12] Yu DS, Lee DT, Man NW (2010). Fatigue among older people: a review of the research literature. Int J Nurs Stud.

[13] Joh DY, Chen LN, Porter G (2014). Proctitis following stereotactic body radiation therapy for prostate cancer. Radiat Oncol.

[14] Chen LN, Suy S, Uhm S (2013). Stereotactic body radiation therapy (SBRT) for clinically localized prostate cancer: the Georgetown University experience. Radiat Oncol.

[15] Flink H, Bergdahl M, Tegelberg A, Rosenblad A, Lagerlöf F (2008). Prevalence of hyposalivation in relation to general health, body mass index and remaining teeth in different age groups of adults. Community Dent Oral Epidemiol.

[16] Beetz I, Schilstra C, van der Schaaf A (2012). NTCP models for patient-rated xerostomia and sticky saliva after treatment with intensity modulated radiotherapy for head and neck cancer: the role of dosimetric and clinical factors. Radiother Oncol.

[17] Taïeb D, Foletti JM, Bardiès M, Rocchi P, Hicks RJ, Haberkorn U (2018). PSMA-targeted radionuclide therapy and salivary gland toxicity: why does it matter?. J Nucl Med.

[18] Calais J, Czernin J, Thin P (2021). Safety of PSMA-targeted molecular radioligand therapy with (177)Lu-PSMA-617: results from the prospective multicenter phase 2 trial RESIST-PC (NCT03042312). J Nucl Med.

[19] Ramnaraign B, Sartor O (2023). PSMA-targeted radiopharmaceuticals in prostate cancer: current data and new trials. Oncologist.

[20] Devlin NJ, Shah KK, Feng Y, Mulhern B, van Hout B (2018). Valuing health-related quality of life: an EQ-5D-5L value set for England. Health Econ.

[21] Potosky AL, Ahn J, Xia Y (2024). Demographic and clinical factors associated with health-related quality-of-life profiles among prostate cancer survivors. JCO Oncol Pract.

